# The Prognostic Role of STEAP1 Expression Determined via Immunohistochemistry Staining in Predicting Prognosis of Primary Colorectal Cancer: A Survival Analysis

**DOI:** 10.3390/ijms17040592

**Published:** 2016-04-19

**Authors:** Ching-Hsiao Lee, Sung-Lang Chen, Wen-Wei Sung, Hung-Wen Lai, Ming-Ju Hsieh, Hsu-Heng Yen, Tzu-Cheng Su, Yu-Hu Chiou, Chia-Yu Chen, Cheng-Yu Lin, Mei-Ling Chen, Chih-Jung Chen

**Affiliations:** 1 Department of Medical Technology, Jen-Teh Junior College of Medicine, Nursing and Management, Miaoli 356, Taiwan; heecs@ms38.hinet.net (C.-H.L.); flutewayne@gmail.com (W.-W.S.); 2 School of Medicine, Chung Shan Medical University, Taichung 402, Taiwan; cshy650@csh.org.tw (S.-L.C.); 91646@cch.org.tw (H.-H.Y.); 3 Department of Urology, Chung Shan Medical University Hospital, Taichung 402, Taiwan; 4 Department of Medical Education, Chung Shan Medical University Hospital, Taichung 402, Taiwan; 5 Institute of Medicine, Chung Shan Medical University, Taichung 402, Taiwan; 170780@cch.org.tw (M.-J.H.); bearking7215@hotmail.com (Y.-H.C.); 6 Department of Surgery, Changhua Christian Hospital, Changhua 500, Taiwan; 143809@cch.org.tw; 7 School of Medicine, National Yang Ming University, Taipei 112, Taiwan; 8 Cancer Research Center, Changhua Christian Hospital, Changhua 500, Taiwan; 9 School of Optometry, Chung Shan Medical University, Taichung 402, Taiwan; 10 Department of Gastroenterology, Changhua Christian Hospital, Changhua 500, Taiwan; 11 Department of Surgical Pathology, Changhua Christian Hospital, Changhua 500, Taiwan; 140062@cch.org.tw (T.-C.S.); 160139@cch.org.tw (C.-Y.C.); 157030@cch.org.tw (C.-Y.L.)

**Keywords:** STEAP1, prognosis, colorectal cancer

## Abstract

STEAP1 (six transmembrane epithelial antigen of the prostate 1) is a transmembrane protein that functions as a potential channel or transporter protein. It is overexpressed in certain cancers and is viewed as a promising therapeutic target. However, the prognostic role of STEAP1 is still controversial, and no role for STEAP1 has yet been indicated in colorectal cancer. The aim of this study was to investigate the possible association of STEAP1 expression with colorectal cancer prognosis. STEAP1 expression was analyzed by immunohistochemical staining of a tissue array of 165 cancer specimens from primary colorectal cancer patients. The mean and medium follow-up times after surgery were 5.1 and 3.9 years, respectively. A total of 139 patients died during the 13 years of follow-up in the survey period. The prognostic value of STEAP1 with respect to overall survival was analyzed by Kaplan-Meier analysis and Cox proportional hazard models. In total, 164 samples displayed detectable STEAP1 expression in the cytoplasm and membrane. Low STEAP1 expression was correlated with poor overall survival (five-year survival: 33.7% *vs.* 57.0%, low expression *vs.* high expression, *p* = 0.020). Accordingly, multivariate analysis identified low STEAP1 expression as an independent risk factor (hazard ratio = 1.500, *p* = 0.018), especially in elderly patients or those with late stage cancers, late *T* values, and early *N* values. We suggest that analysis of STEAP1 expression by immunohistochemical staining could serve as an independent prognostic marker for colorectal patients. This finding should be validated by other investigative groups.

## 1. Introduction

A patient’s tumor stage at diagnosis is a key determinant of cancer prognosis and response to treatments [1]. Tumor metastasis and recurrence also negatively affect patient prognosis and quality of life [2]. Therefore, the identification of prognostic indicators that can identify cancers at early stages has become a key area of cancer research. We recently observed that STEAP1 (six transmembrane epithelial antigen of the prostate 1) is overexpressed at the RNA level in circulating colorectal tumor cells (data not published), which raises the possibility that STEAP1 might be associated with tumor metastasis. The present study therefore investigated the potential usefulness of STEAP1 in the prognosis of colorectal cancer patients.

The STEAP family of proteins consists of four members: STEAP1–STEAP4 [3]. STEAP1, a 6-transmembrane protein, is expressed in normal tissues, but its expression is most abundant in prostate tissue [3,4]. It acts as an ion channel or transporter protein in tight junctions and gap junctions, or it can function in cell adhesion and intercellular communication [5]. A role in cell proliferation or anti-apoptosis is also indicated, as blocking STEAP1 with a monoclonal antibody induces cell apoptosis [5].

Analysis of the transcriptomes and proteomes of Ewing’s tumors, together with functional studies, has also revealed a correlation between STEAP1 expression and oxidative stress responses, including elevation of levels of reactive oxygen species that promote expression of proinvasive genes [6]. The STEAP antigens from prostate and melanoma tumor cells also induce cytotoxic and helper T lymphocytes, suggesting that the STEAP protein might be useful as an antitumor peptide vaccine for eradication of STEAP-expressing tumors [7,8]. STEAP1 therefore appears to have as yet unconfirmed roles in tumor malignancy and may therefore represent a potential target for therapeutic strategies.

STEAP1 is much more strongly expressed in tumor cells than in normal tissues and its expression is associated with the malignant phenotype of several types of cancers, including prostate cancer, head and neck squamous cell carcinoma, Ewing family tumors, breast cancer, and melanoma [3,9,10,11,12,13]. STEAP1 has also been identified as a prognostic marker in Ewing’s sarcoma and prostate carcinoma [11,14,15]. Nevertheless, its prognostic role remains controversial [13].

High STEAP1 expression in prostate cancer patients is associated with poor prognosis [9,15]. High STEAP1 mRNA expression in some Ewing family tumors can be associated with cancer metastasis and short survival [11]; however, other Ewing’s sarcoma patients with high STEAP1 protein expression show associated good clinical outcomes [14].

These discrepancies complicate the establishment of a role for STEAP1 in predicting clinical outcome or its use as a therapeutic target. In addition, no study has yet provided evidence for a role for STEAP1 in colorectal cancer. The present investigation therefore explored the possibility of a potential prognostic role for STEAP1 in colorectal cancer.

## 2. Results

### 2.1. STEAP1 Is Expressed in the Majority of Colorectal Cancer Specimens and Locates to the Cytoplasm and Membrane

**Figure 1:**
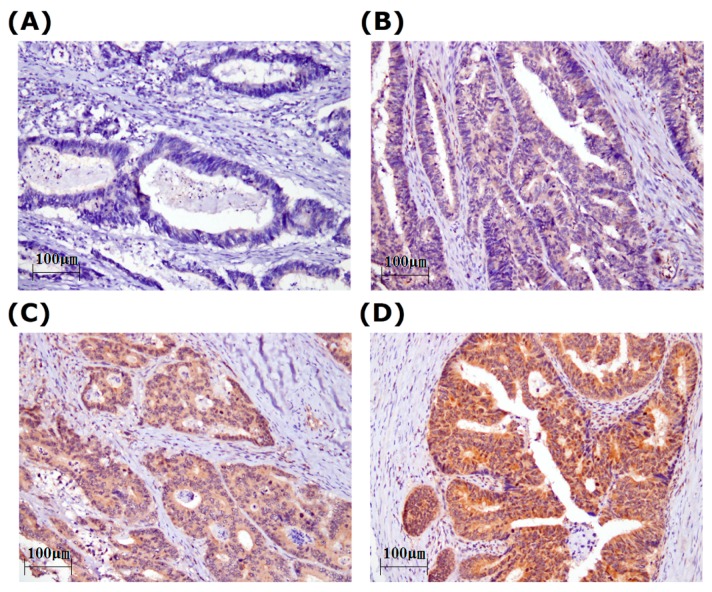
Representative immunostaining of STEAP1 in colorectal cancer in tissue arrays. STEAP1 expression scores were (**A**) 0; (**B**) 100; (**C**) 200; (**D**) 300.

We verified a role for STEAP1 in colorectal cancer patients by evaluating its expression in primary tumors from 165 patients. Table 1 and Supplementary Materials Table S1 show the clinicopathological characteristics of the study subjects. Their mean age was 64.2 ± 13.4 years (mean ± SD) and the gender ratio was 0.76:1.00 (female:male). In total, 23 patients had stage I, 63 had stage II, 51 had stage III, and 28 had stage IV tumors. 27 patients had metastasis at diagnosis. STEAP1 expression was evaluated by Immunohistochemical (IHC) staining of tissue arrays. Figure 1A–D shows a representative staining for STEAP1 in colorectal tumor specimens. The membranocytoplasmic and nucleic expressions of STEAP1 were scored separately by pathologists. However, no STEAP1 expression was observed in the nuclei in any specimen. In total, 164 (99.4%) specimens showed STEAP1 expression in the cytoplasm and membrane. The mean and median STEAP1 expression scores were 122.6 ± 65.1 and 100, respectively; we used 100 as a cut-off point for further analysis. We defined STEAP1 expression >100 as high expression and STEAP1 expression ≤100 as low expression. We further evaluated the relationships between STEAP1 expression and clinicopathological characteristics, but found no significant difference between gender, stage, grade, tumor location, and TNM value (Table 1 and Supplementary Materials Table S1). STEAP1 was strongly expressed in the cytoplasm and membranes, but not in the nuclei, of cancer cells.

### 2.2. The Prognostic Role of STEAP1 Expression in Colorectal Cancer Patients

**Figure 2:**
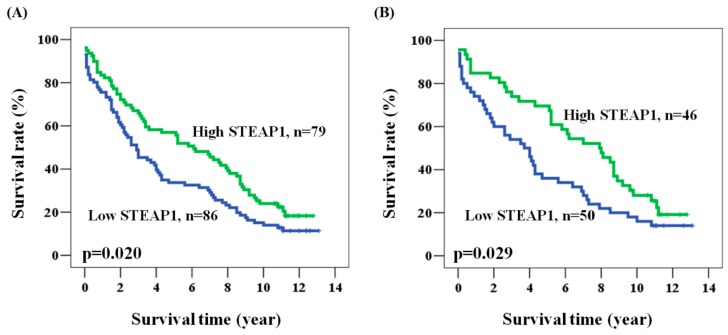
Kaplan-Meier actuarial analysis of STEAP1 expression in overall survival of patients with (**A**) all subjects; and (**B**) N0.

We verified a prognostic role for STEAP1 expression in colorectal cancer by collecting overall survival data from 165 patients. The mean and median follow-up times after surgery were 5.4 and 3.9 years (ranging from 0.1 to 13.1 years), respectively. During the 13 years of follow-up in this survey, 139 (84.2%) patients died and the five-year survival rate was 44.8%. We used the Cox regression model to evaluate the prognostic role of age, gender, stage, and STEAP1 expression in colorectal cancer patients. Among the clinicopathological characteristics, late stage (stage II + III + IV) was significantly associated with poor clinical outcome (hazard ratio (HR) = 1.797, 95% confidence interval (CI) = 1.079–2.992, *p* = 0.024 for univariate analysis; HR = 1.851, 95% CI = 1.111–3.085, *p* = 0.018 for multivariate analysis, Table 2 and Table 3). Overall survival was shorter for patients with late stage than with early stage cancer (five-year survival: 40.1% *vs.* 73.9%, stage II + III + IV *vs.* I, *p* = 0.024). However, age and gender showed no significant association with patient prognosis (Table 2 and Table 3). Interestingly, prognosis was significantly poorer in patients with low STEAP1 expression than with high STEAP1 expression (HR = 1.477, 95% CI = 1.056–2.066, *p* = 0.023 for univariate analysis; HR = 1.500, 95% CI = 1.071–2.100, *p* = 0.018 for multivariate analysis; five-year survival: 33.7% *vs.* 57.0%, low STEAP1 *vs.* high STEAP1, *p* = 0.020, Table 2 and Table 3, and Figure 2A). We also investigated the influence of tumor grade and histology on the prognostic role of STEAP1 by further adjusting for tumor grade, histology, age, gender, and stage. The adjusted results confirmed that low STEAP1 expression still predicted poor prognosis (HR = 1.592, 95% CI = 1.126–2.251, *p* = 0.009 for multivariate analysis). These findings suggested a more favorable prognosis for colorectal cancer patients with high STEAP1 expression.

### 2.3. Prognostic Role of STEAP1 Expression according to the Clinicopathological Characteristics of Colorectal Cancer

We identified the patient characteristics that favored the use of STEAP1 as a prognostic marker by analyzing the clinical outcome of STEAP1 expression according to clinicopathological characteristics. Table 4 shows a significant association of STEAP1 expression, as analyzed in a multivariate model, with the prognosis in male patients, in patients aged ≥65, and in patients with late stage cancer, a late *T* value, or an early *N* value. Kaplan-Meier analysis further confirmed the prognostic value of these characteristics for colorectal cancer (*p* values: 0.025, 0.007, 0.020, 0.013 and 0.029; patients of age ≥65, male, late stage, late *T* value, and early *N* value, respectively). Interestingly, patients with a late *T* or early *N* value showed high and significant hazard ratios (HRs) for STEAP1 (multivariate analysis: HR = 1.602, 95% CI = 1.114–2.303, *p* = 0.011 for patients with late T values; HR = 1.718, 95% CI = 1.097–2.691, *p* = 0.018 for patients with an early *N* value, Table 4 and Figure 2B). These results confirmed a significant prognostic value for STEAP1 in patients with specific clinicopathological characteristics.

## 3. Discussion

To the best of our knowledge, this is the first study to report a potential prognostic role for STEAP1 in colorectal cancer. We recently found STEAP1 overexpression at the RNA level in circulating tumor cells of colorectal cancer, which suggested that STEAP1 might have a role in tumor metastasis. Interestingly, in the present study, low expression of STEAP1 is associated with poor prognosis in our population (Table 3 and Figure 2A). The underlying mechanism whereby STEAP1 promotes circulating tumor cell formation and favorable prognosis needs further investigation.

The prognostic role of STEAP1 was more significant in elderly patients, and in those with late stage cancers, late *T* values, and early *N* values (Table 4). The IHC staining of STEAP1 protein in colorectal tumor tissue revealed no nuclear expression of STEAP1, and at least 99% of the specimens had membranocytoplasmic STEAP1 expression (Figure 1A–D). These staining results fit with the biological properties and with the previously reported results from studies on different types of cancer, where STEAP1 was detected in the tumor portion but not in adjacent parts of the specimens [3,9,10,11,12,13]. No correlation was found between the clinicopathological characteristics and STEAP1 expression in our study (Table 1). Previous studies have suggested an association between STEAP1 and the malignant phenotype of cancer cells [3,9,10,11,12,13]. These contradictory results could indicate a variation in the role of STEAP1, depending on the type of cancer.

A prognostic role has not been previously demonstrated for STEAP1 in colorectal cancer. The overexpression of STEAP1 in tumor tissue seems to be associated with tumor malignancy, advanced grade, and poor prognosis [9,10,14,15]. However, the prognostic role that STEAP1 plays in various other types of cancer remains controversial. For example, prostate cancer patients with STEAP1 overexpression in the tumor portion show significantly poor survival [15]. Similarly, Ewing family tumors with mRNA expression of a combination of several markers, including STEAP1, also indicate an association between high STEAP1 expression and poor patient survival [11]. However, the findings are not conclusive, as still other studies show an association between STEAP1 expression and improved prognosis. One possibility is that the effects of STEAP1 expression might differ depending on the cancer cell types or populations. The contradictory results seen for Ewing’s sarcoma patients indicate the existence of specific subgroups of patients, some of which might benefit from adapted therapy regimens for treatment of Ewing’s sarcoma [14].

In the present study, STEAP1 expression was detected mainly in colorectal tumor tissue. After adjustment for potential confounders, we determined that patients with low STEAP1 expression in the tumor portion had significantly poor prognosis (Table 3). This finding might not be supported by the known biological function of STEAP1, but the favorable prognostic value of high STEAP1 expression is the same as previously reported for some Ewing's sarcoma patients [14]. Further analysis showed that STEAP1 had a more significant association with clinical outcome in elderly patients and in patients with late stage cancers, late *T* values, and early *N* values (Table 4). This indicated that the prognostic role of STEAP1 might differ among patients with specific clinicopathological characteristics (Table 4).

The small sample size from a single center is the major limitation of the present study. This study also only evaluated overall survival but not relapse-free survival or recurrence-free survival. The double blinding of the IHC score evaluations and statistical analysis to assure accuracy and reduce bias also meant that IHC staining could not be performed until the tumor specimen was obtained. In addition, as with the other studies on the prognostic role of STEAP1, we could not determine any underlying mechanism that would explain why patients with high STEAP1 expression would have favorable clinical outcomes. Further investigation of the possible mechanism of STEAP1 in colorectal cancer is necessary to identify the role of STEAP1 among different cancer types.

## 4. Materials and Methods

### 4.1. Study Subjects and Ethics Statement

The study was approved by the Institutional Review Board and the Ethics Committee of the Changhua Christian Hospital, Changhua, Taiwan (IRB No. 121008, 5 December 2012). We analyzed the data anonymously. The Institutional Review Board and the Ethics Committee of the Changhua Christian Hospital agreed to waive the informed consent from the participants. This study investigated de-linked tissue specimens from 165 primary colorectal cancer patients. All of the patients in this study are not subjected to chemotherapy or radiotherapy before surgery. Surgically resected tumor tissues were collected from patients with confirmed histological diagnosis at Changhua Christian Hospital between 1997 and 2000. Cancers were staged according to the AJCC Colon Cancer Staging, 7th edition (2010). Clinical data were obtained from previous data base anonymously, including gender, age, stage, *T*, *N*, and *M* stages, and follow-up information.

### 4.2. Immunohistochemical Staining and Evaluation of STEAP1 Immunoreactivity

IHC staining was performed at the Department of Surgical Pathology, Changhua Christian Hospital, as previously described [16,17]. Anti-human STEAP1 antibody (1:200 dilution; sc-25514, Santa Cruz Biotechnology, Santa Cruz, CA, USA) was used as used in previous studies [6,14]. Immunoreactivity scores were analyzed by three pathologists using scores defined as previously described [17,18]. The pathologists were blinded to the prognostic data during this study. A final agreement was obtained for each score by using a multiheaded microscope (Olympus BX51 10 headed microscopes, Olympus Corporation, Tokyo, Japan). Normal prostate tissues and prostatic cancer tissues were used as positive controls. Briefly, the cell staining intensity was defined as 0 to 3 (0 = nil; 1 = weak; 2 = moderate; and 3 = strong). The immunoreactivity scores were defined with the cell staining intensity multiplied by the percentage of stained cells which leads to scores from 0 to 300.

### 4.3. Statistical Analysis

The χ^2^ test was applied for discrete or continuous data. The associations between the clinical outcome and STEAP1 were estimated using the univariate method and Kaplan-Meier analysis which is assessed via log-rank test. We adjusted potential confounders which include age, gender, and stage by Cox regression models of multivariate analysis, and the STEAP1 was fitted as an indicator variable. Overall survival time was defined as the interval between the date of surgery and the date of the last follow-up or death. Statistical analyses were conducted via SPSS statistical software (version 15.0) (SPSS, Inc., Chicago, IL, USA). Two-sided, and values of *p* < 0.05 were considered to be statistically significant.

## 5. Conclusions

The present study demonstrates the feasibility of using STEAP1 to predict the prognosis of colorectal cancer patients. Our results suggest that STEAP1 might have a prognostic role, but that this role might depend on the clinicopathological characteristics of particular cancer patients. However, confirmation of this suggestion requires additional study, and the findings presented here require further validation by other investigative groups.

## Figures and Tables

**Table 1:** Relationships of STEAP1 expression with clinical parameters in colorectal cancer patients.

Parameters	Case Number	STEAP1 Expression, *n* (%)		*p* Value ^1^
		Low	High	
Age (year, mean ± SD)		65.1 ± 12.7	63.2 ± 14.2	0.365
Gender				
Female	71	36 (50.7)	35 (49.3)	0.752
Male	94	50 (53.2)	44 (46.8)	
Stage				
I	23	10 (43.5)	13 (56.5)	0.690
II	63	35 (55.6)	28 (44.4)	
III	51	25 (49.0)	26 (51.0)	
IV	28	16 (57.1)	12 (42.9)	
*T* value				
1	6	4 (66.7)	2 (33.3)	0.201
2	20	7 (35.0)	13 (65.0)	
3	123	64 (52.0)	59 (48.0)	
4	16	11 (68.8)	5 (31.3)	
*N* value				
0	96	50 (52.1)	46 (47.9)	0.994
1	63	33 (52.4)	30 (47.6)	
2	6	3 (50.0)	3 (50.0)	
*M* value				
0	138	70 (50.7)	68 (49.3)	0.417
1	27	16 (59.3)	11 (40.7)	

^1^ *p* Value was calculated by χ^2^ test.

**Table 2:** Univariate analysis of the influence of various parameters on overall survival in colorectal cancer patients.

Parameter	Category	Overall Survival			
		5-Year Survival (%)	HR	95% CI	*p* Value
Age, year	≥65/<65	43.3/47.1	1.328	0.943–1.870	0.104
Gender	Male/Female	44.7/45.1	1.161	0.828–1.627	0.387
Stage	II + III + IV/I	40.1/73.9	1.797	1.079–2.992	0.024
STEAP1 expression	Low/High	33.7/57.0	1.477	1.056–2.066	0.023

**Table 3:** Multivariate analysis of the influence of various parameters on overall survival in colorectal cancer patients.

Parameter	Category	Overall Survival ^1^			
		5-Year Survival (%)	HR	95% CI	*p* Value
Age, year	≥65/<65	43.3/47.1	1.404	0.994–1.984	0.054
Gender	Male/Female	44.7/45.1	1.246	0.886–1.752	0.207
Stage	II + III + IV/I	40.1/73.9	1.851	1.111–3.085	0.018
STEAP1 expression	Low/High	33.7/57.0	1.500	1.071–2.100	0.018

^1^ Adjusted for age, gender, and stage.

**Table 4:** Multivariate analysis of the influence of STEAP1 expression in subgroups according to clinical parameters on overall survival in colorectal cancer patients.

Parameter	Overall Survival ^1^			
	5-Year Survival (%)	HR	95% CI	*p* Value
All cases	33.7/57.0	1.500	1.071–2.100	0.018
Age (year)				
<65	34.3/60.6	1.284	0.743–2.219	0.370
≥65	33.3/54.3	1.597	1.034–2.465	0.035
Gender				
Female	38.9/51.4	1.116	0.662–1.884	0.680
Male	30.0/61.4	1.756	1.123–2.745	0.014
Stage				
I	70.0/76.9	1.365	0.514–3.627	0.532
II + III + IV	28.9/53.0	1.545	1.077–2.216	0.018
*T* value				
1 + 2	72.7/73.3	1.134	0.440–2.924	0.795
3 + 4	28.0/53.1	1.602	1.114–2.303	0.011
*N* value				
0	36.0/69.6	1.718	1.097–2.691	0.018
1 + 2	30.6/39.4	1.264	0.751–2.129	0.378
*M* value				
0	37.1/63.2	1.641	1.130–2.383	0.009
1	18.8/18.2	0.789	0.305–2.038	0.624

^1^ Adjusted for age, gender, and stage.

## Supplementary Materials

Supplementary materials can be found at http://www.mdpi.com/1422-0067/17/4/592/s1.
