# Supplementary material for: The Prognostic Role of STEAP1 Expression Determined via Immunohistochemistry Staining in Predicting Prognosis of Primary Colorectal Cancer: A Survival Analysis
Source: Int J Mol Sci. 2016 Apr 19;17(4):592. doi: 10.3390/ijms17040592 (PMC4849046; doi:10.3390/ijms17040592)
Supplement: Supplementary file 1 [file ijms-17-00592-s001.pdf]

Supplementary Materials: The Prognostic Role of STEAP1 Expression Determined via Immunohistochemistry Staining in Predicting Prognosis of Primary Colorectal Cancer: A Survival Analysis

Ching-Hsiao Lee, Sung-Lang Chen, Wen-Wei Sung, Hung-Wen Lai, Ming-Ju Hsieh, Hsu-Heng Yen, Tzu-Cheng Su, Yu-Hu Chiou, Chia-Yu Chen, Cheng-Yu Lin, Mei-Ling Chen and Chih-Jung Chen

Table S1. Relationships of STEAP1 expression with pathological parameters in colorectal cancer patients.

Parameters	Case Number	STEAP1 Expression		p Value
		Low	High	
Tumor site				
Ascending	15	5 (33.3)	10 (66.7)	0.678
Transverse	1	0 (0.0)	1 (100.0)	
Descending	8	5 (62.5)	3 (37.5)	
Rectum	58	29 (50.0)	29 (50.0)	
Sigmoid	26	11 (42.3)	15 (57.7)	
Rectosigmoid	10	5 (50.0)	5 (50.0)	
Cecum	4	1 (25.0)	3 (75.0)	
Missing	43	30 (69.8)	13 (30.2)	
Histology				
Non-mucinous	154	84 (54.5)	70 (45.5)	0.034
Mucinous	10	2 (20.0)	8 (80.0)	
Missing	1	0 (0.0)	1 (100.0)	
Grade				
1	5	3 (60.0)	2 (40.0)	0.851
2	149	78 (52.3)	71 (47.7)	
3	11	5 (45.5)	6 (54.5)	
